# Rating the quality of teamwork—a comparison of novice and expert ratings using the Team Emergency Assessment Measure (TEAM) in simulated emergencies

**DOI:** 10.1186/s13049-019-0591-9

**Published:** 2019-02-08

**Authors:** Julia Freytag, Fabian Stroben, Wolf E. Hautz, Stefan K. Schauber, Juliane E. Kämmer

**Affiliations:** 1 Simulated Patients Program, Office of the Vice Dean for Teaching and Learning, Charité – Universitätsmedizin Berlin, corporate member of Freie Universität Berlin, Humboldt-Universität zu Berlin, and Berlin Institute of Health, Charitéplatz 1, 10117 Berlin, Germany; 2 Lernzentrum, Office of the Vice Dean for Teaching and Learning, Charité – Universitätsmedizin Berlin, corporate member of Freie Universität Berlin, Humboldt-Universität zu Berlin, and Berlin Institute of Health, Charitéplatz 1, 10117 Berlin, Germany; 3 AG Progress Test Medizin, Charité – Universitätsmedizin Berlin, corporate member of Freie Universität Berlin, Humboldt-Universität zu Berlin, and Berlin Institute of Health, Charitéplatz 1, 10117 Berlin, Germany; 4 Department of Emergency Medicine, Inselspital, Bern University Hospital, University of Bern, Freiburgstrasse 4, 3010 Bern, Switzerland; 5 Centre for Health Sciences Education, University of Oslo, Gaustadalléen 30, 0373 Oslo, Norway; 6 Center for Adaptive Rationality, Max Planck Institute for Human Development, Lentzeallee 94, 14195 Berlin, Germany

**Keywords:** Teamwork, Non-technical skills, Expert rater, Novice rater, Assessment, Simulation, Resuscitation, Emergency

## Abstract

**Background:**

Training in teamwork behaviour improves technical resuscitation performance. However, its effect on patient outcome is less clear, partly because teamwork behaviour is difficult to measure. Furthermore, it is unknown who should evaluate it. In clinical practice, experts are obliged to participate in resuscitation efforts and are thus unavailable to assess teamwork quality. Consequently, we sought to determine if raters with little clinical experience and experts provide comparable evaluations of teamwork behaviour.

**Methods:**

Novice and expert raters judged teamwork behaviour during 6 emergency medicine simulations using the Teamwork Emergency Assessment Measure (TEAM). Ratings of both groups were analysed descriptively and compared with *U* and *t* tests. We used a mixed effects model to identify the proportion of variance in TEAM scores attributable to rater status and other sources.

**Results:**

Twelve raters evaluated 7 teams rotating through 6 cases, for a total of 84 observations. We found no significant difference between expert and novice ratings for 7 of the 11 items of the TEAM or in the sums of all item scores. Novices rated teamwork behaviour higher on 4 items and overall. Rater status accounted for 11.1% of the total variance in scores.

**Conclusions:**

Experts’ and novices’ ratings were similarly distributed, implying that raters with limited experience can provide reliable data on teamwork behaviour. Novices show a consistent, but slightly more lenient rating behaviour. Clinical studies and real-life teams may thus employ novices using a structured observational tool such as TEAM to inform their performance review and improvement.

**Electronic supplementary material:**

The online version of this article (10.1186/s13049-019-0591-9) contains supplementary material, which is available to authorized users.

## Background

Medical response to high-urgency situations such as cardiac arrest remains an area for improvement. Depending on their initial rhythm, only around 25% of patients with out-of-hospital cardiac arrest achieve a return of spontaneous circulation (ROSC) [1] and overall survival to discharge lies around 10% [1, 2]. Survival of patients with in-hospital cardiac arrest is higher but still only ranges between 18 and 44% [3, 4].

Besides technical skills such as providing an adequate compression rate [5], working effectively together in a team is connected to patient outcome in high-urgency patients; therefore, training in teamwork behaviour^1^ has the potential to improve survival rates [6, 7]. For example, different studies have shown that training in communication and leadership skills in emergency response teams leads to improved ROSC and survival rates [8–10]. Findings from experimental investigations suggest that improved team communication and leadership result in a significant reduction of no-flow time and better chest compressions in simulated resuscitations [6]. Further, working together in teams can improve diagnostic accuracy in emergency medicine [11, 12] as well as the quality of care compared to individual performance [13].

However, what exactly good teamwork behavior is depends on the task and the role of each team member. Generic rules such as “always practice closed-loop communication” are misleading. For example, one study demonstrated that closed-loop communication initiated by the team leader was associated with a shorter time until the correct diagnosis in an emergency trauma case was made, whereas the same communication pattern delayed the decision significantly if initiated by team members [14]. Also, directive leadership behaviour improved technical performance at the beginning of a resuscitation, whereas in later phases, structuring inquiry (e.g., “What do we know about the patient?”) was associated with improved technical performance [6]. These findings show the need to collect more data on teamwork, investigate specific individual and team behaviours, and take differences in task requirements into account. For this, we need valid and reliable tools with known properties that are feasible to use in real-world settings.

In addition, evidence of improvements in patient outcomes as a result of teamwork interventions is limited to a few small studies, many conducted in simulated emergencies [6, 7, 9, 10, 14]. Fung and colleagues suggested that the lack of an objective measurement of team performance is one reason for this paucity of data [15]. While, for example, chest compression rate and depth can nowadays be tracked [16] and technical solutions help to document resuscitations more precisely [17], teamwork behaviour is not easy to measure, especially in real-life situations. Such information is not only relevant for research but also a necessity to inform debriefings after resuscitation [18]. Consequently, different tools have been developed to assess individuals non-technical skills as well as teamwork behaviour. Some of these tools are designed for a specific context, such as the anaesthetists’ non-technical skills behavioural marker system (ANTS) [19] or the observational teamwork assessment for surgery (OTAS) [20–22], others are intended to be more generic and independent of context, such as the Ottawa Crisis Resource Management Global Rating Scale [23].

One tool that has been used in both, real-life emergency situations and simulated emergency trainings, is the Teamwork Emergency Assessment Measure (TEAM) [24–27]. The TEAM was designed for emergency teams and is particularly used to assess teamwork, leadership and task-management in high emergency situations such as resuscitation [24, 28]. Since its development in 2010, TEAM has been translated into French [29], Hebrew and Chinese (available via www.medicalemergencyteam.com) and was used in real-life resuscitations [27, 28] and simulated environments (in centre and in situ) [24, 29–32], observing teams of medical and nursing students [24, 31], nurses and physicians [25, 27, 30, 32] and comparing teams with different levels of expertise [29] (see Additional file 1). A recent review showed that it has good psychometric properties in contrast to most other tools for assessing teamwork [18]. In summary, the TEAM has been used in several clinical and simulation-based studies with comparable outcomes (see Additional file 1) and is the most appropriate and valid tool for evaluating teamwork in emergency teams.

While some of the tools meant to quantify non-technical skills and teamwork are intended to be used as self-assessments by practitioners and trainees alike (such as the Mayo High Performance Teamwork Scale [33]), all of the above were designed for raters external to the team they observe [19, 22–24]. Selecting raters to use such instruments is as important as having a suitable tool, yet empirical evidence is lacking concerning *who* should or can assess teamwork behaviour in real or simulated emergencies. During training, it is usually the task of expert raters to assess and debrief participants [34, 35]. Until now, most studies using TEAM have employed expert raters; in two cases TEAM was used as a self-rating instrument for experienced team members as logistical reasons did not allow to recruit external observers [25, 27]. In practice, it might be even harder to find raters with high clinical expertise to observe resuscitations because of their high workload. Such an approach would also lead to ethical problems—especially given that expert raters would have broad knowledge of teamwork and emergency medicine (making them expert in this area), but would be restricted to observing. A possible solution for this methodological, ethical, and organisational dilemma could be the use of less clinically experienced raters, such as residents [36, 37].

We therefore compared novices with expert raters, as these two groups represent the widest difference in clinically relevant qualifications. Both types of raters evaluated teamwork behaviour in an extensive emergency simulation using TEAM. Equivalent ratings from the two rater groups would justify ratings by less experienced raters such as residents also in the workplace.

## Methods

### Description and translation of TEAM

TEAM consists of 11 items measuring the teamwork behaviour of medical teams dealing with critical situations [24]. The tool consists of 3 subscales: leadership (2 items), teamwork (7 items), and task management (2 items); all items are rated on a Likert scale of 0 (*never/hardly ever*) to 4 (*always/nearly always*). A sum score with a possible range of 0 to 44 can be calculated. Furthermore, overall performance is rated on a global rating scale (GRS) of 1 to 10.

Although a French version exists that confirmed the excellent psychometric properties of the original English version [29], a German version of TEAM is currently lacking. Addressing this gap, our research team has translated TEAM into German using the TRAPD (translation, review, adjudication, pre-testing, and documentation) methodology [38]. A pre-study was conducted to check feasibility and inter-rater reliability and showed excellent results [39].

### Data collection

The study was conducted at Charité Universitätsmedizin Berlin during an emergency medicine simulation for final year medical students [40]. During this simulation, the participants acted in teams of 5 and rotated through 6 cases (duration about 30 min each; see Additional file 2: Table S2 for details), in which they had to deal with common emergencies including 1 resuscitation. These cases were realized using simulated patients and high-fidelity simulation. For every case, 1 participant was declared team leader; leadership changed after every case.

### Raters

Two groups of raters, one of novices and one of content experts, evaluated participants’ teamwork behaviour throughout each case. For the novice raters, we recruited tutors from the local skills lab. They were advanced medical students with emergency medicine experience through clinical electives and/or work experience as paramedics. Expert raters were physicians and psychologists with broad experience in emergency medicine and/or expertise in rating and teaching teamwork during simulation-based education.

Before using TEAM to rate the teams’ performances, all raters participated in a rater training [39], which included an introduction to TEAM as a rating instrument, information about common rating errors, and a frame-of-reference training, where videotaped examples of teamwork were rated and discussed [41]. Novice and expert raters received the same training (same length, content etc.) Due to organisational reasons they were trained on two separate occasions. Neither the experts nor the novices had any previous experience with the TEAM as a rating instrument.

### Data analysis

Data were analysed using SPSS 24 (Armonk, NY: IBM Corp.) and R, version 3.4.4 [42]. Different descriptive measures were computed separately for the ratings given by novice and expert raters. To analyse the measurement properties of the German version of TEAM, we calculated its’ reliability (Cronbach’s α), the item-total-score correlation and the correlation of all items plus the sum score with the GRS. As a measure of construct validity, we conducted a principal component analysis (PCA). In a PCA, the objective is to analyse the structure of a data set and to combine a number of observed variables into one factor. We used PCA to check if the items of the German TEAM could be combined into one general component, as was shown for the original version [24, 25]. All results were compared to other studies using TEAM.

Inter-rater reliability between novice and expert raters was calculated (using the intraclass correlation coefficient, ICC) to explore the agreement between these 2 groups. Additionally, their ratings were compared using Mann–Whitney *U* tests (for the 11 single items) and *t* tests (for the sum score and GRS).

We used a mixed effects model to identify the sources of variance in TEAM’s global rating scale [43]. Mixed effects models are an extension of the ordinary linear regression model that allow for estimating one or more variance components (i.e., random effects) in addition to the residual variance term. In this study, we estimated variance components for teams, raters, rater status (novice or expert), cases, and their first-order interactions.

## Results

### Participants

During our 8-h emergency simulation, 12 raters (6 novices, 6 experts) rated 7 teams rotating through 6 cases each, resulting in 84 observations in total. Each team consisted of 5 participants; their age ranged between 22 and 46 years (mean [*M*] = 26.5 years, standard deviation [*SD*] = 4); 46.9% of the participants were female. The team’s performance was rated by pairs of independent observers, 1 expert and 1 novice rater. Both of them were present while the simulation took place and independently rated the teamwork right afterwards. The novice raters had between 1 and 2.5 years of experience in student-assisted learning; experts (5 physicians and 1 psychologist) had 3.5 to 10 years of experience in teaching, including facilitating medical simulations. Further information about the characteristics of the novice and expert raters can be found in Table 1.

**Table 1:** Characteristics of the novice and expert raters

	Novice raters	Expert raters
N	6	6
Age (Median)	20–33 (24)	26–37 (31.5)
Profession	medical students	5 medical doctors, 1 psychologist
Teaching experience	1–2.5 years (student-assisted learning)	3.5 to 10 years (clinical teaching, simulation-based education, faculty development)
Clinical expertise	Internships (up to 120 days)	1–10 years

### Measurement properties of the German translated version of TEAM

We report the measurement properties of the German translated version of TEAM in terms of (1) reliability, (2) item-total-score correlation (i.e., discrimination) and (3) correlation of individual items and the TEAM sum score to the GRS. First, reliability of TEAM instrument—calculated separately for each case and independently for expert and novice raters—had a mean Cronbach’s alpha of 0.89 (*SD* = 0.06) for experts and a mean Cronbach’s alpha of 0.85 (*SD* = 0.19) for novices. For expert raters, the lowest alpha was .79; it was observed on the case 1 (discipline: surgery). The lowest alpha for novice raters was observed on case 5 (discipline: anaesthesia; alpha = .47). Second, items generally were positively correlated to the sum score of TEAM with a mean of *M*_corr(experts)_ = 0.71 (*SD* = 0.09) and *M*_corr(novices)_ = 0.69 (*SD* = 0.17) across cases for experts and novices, respectively. Third, the TEAM items and the GRS score showed a mean correlation of *M*_*c*orr(experts)_ = 0.71 (*SD* = 0.10) for experts and *M*_*c*orr(novices)_ = 0.69 (*SD* = 0.17) for novices. Finally, across stations, the TEAM sum score and the GRS were significantly correlated both for experts (*r* = 0.90, *p* < .001) and novices (*r* = 0.85, *p* < .001). All psychometric properties mentioned above are compared to the data of studies with the English and French versions of TEAM in Table *2*.

**Table 2:** Psychometric properties of the German, the French, and the original English version of TEAM [24, 25, 27, 29–32, 54]

Measurement	English TEAM	French TEAM	German TEAM expert rating	German TEAM novice rating
Cronbach’s α	0.78–0.97	0.95	0.93	0.94
Inter-item correlation (Spearman’s rho)	0.21–1	0.47–0.85	0.29–0.73	0.42–0.75
Item–total correlation	0.42–0.94	0.64–0.79	0.59–0.81	0.38–0.81
Inter-rater reliability^a^ (ICC)	0.60–0.94	0.93	0.66	

*Legend*: *TEAM* Teamwork Emergency Assessment Measure, *ICC* intraclass correlation coefficient

^a^The French and German ICC represent the ICC of the sum score; the range for the ICC in studies with the original TEAM contains both ICC of sum scores and mean ICC of the 11 TEAM items

### Combination of TEAM items into a general component

We conducted the PCA to examine to which degree the individual TEAM items could be combined into a general component. Prior to conducting the PCA, the adequacy of the observed correlation matrix was evaluated using three related statistical criteria. First, the range of inter-item correlations was range_*r*;expert_ = 0.29–0.73 and range_*r;*novices_ = 0.42–0.75. Second, the Kaiser–Meyer–Olkin (KMO) criterion summarizes in how far the obtained variables share unique variance and thus might be combined into a single factor. The KMO was 0.87 for both, expert and novice ratings and therefore exceeded the commonly recommended cut-off of 0.6. Third, the Bartlett test of sphericity which was statistically significant (*p* < 0.001) for both experts and novices, suggesting that the correlation matrix is different from an identity matrix (that is, a correlation matrix where only auto-correlations in the diagonal are of substantial magnitude).

Taken together, the items in the TEAM were sufficiently inter-related to conduct a PCA. The according PCA was, again, conducted independently for novice and expert raters. Results were largely comparable since for both, experts and novices, a dominant first component was found which explained 59 and 65% of the observed variance, respectively.

### Agreement between expert- and novice-based ratings

We calculated the inter-rater reliability between novice and expert raters based on the sum scores of TEAM and found an intra-class correlation of ICC = 0.66 (considered *moderate* [44] to *good* [45]). This resemblance between the ratings is also reflected by the finding that expert and novice raters agreed by and large on the lowest and best performing groups for a given case. That is, ratings of experts and novices were consistent in 75% of cases when comparing which teams received the 2 highest and the 2 lowest scores for each case. Furthermore, ratings of experts and novices were compared on the item-level using *U*-tests. On 7 of 11 items, no statistically significant difference was found (items 1, 4, 5, 6, 7, 9, 11; *p* = .06–.86). However, on 4 of 11 items novices rated teamwork behaviour higher than experts on average (items 2, 3, 8, 10; *p* = .04–.004). Furthermore, across cases, we found no statistically significant difference between the TEAM sum scores for experts and novices (*M*_novice_ = 30.4, *SD*_novice_ = 8.6, *M*_expert_ = 27.0, *SD*_expert_ = 8.4; *t*(82) = 1.8, *p* = .08). Finally, for the GRS, we found that novices (*M*_novice_ = 7.1, *SD*_novice_ = 1.6) gave generally higher ratings as compared to experts (*M*_expert_ = 6.1, *SD*_expert_ = 1.9). This difference was statistically significant with *t*(82) = 2.5 and *p* = .02.

**Fig. 1:**
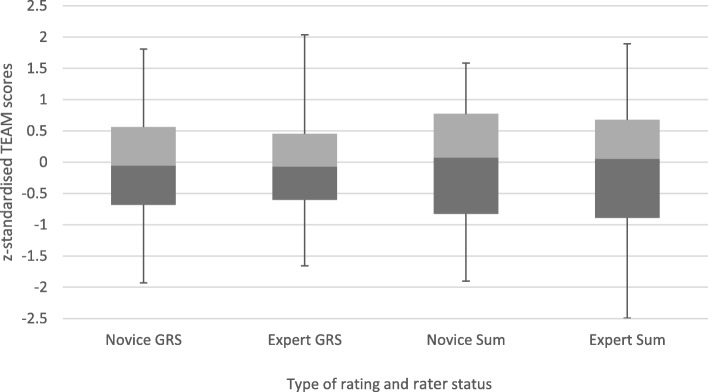
Distribution of standardised global rating scale (GRS) and sum scores of novice and expert raters. *Legend:* Quartiles 1 and 2 are shown as dark grey boxes, quartiles 3 and 4 as light grey boxes; Whiskers show the minimum and maximum scores. TEAM = Teamwork Emergency Assessment Measure

Further details on the differences and similarities in ratings between experts and novices are given in Fig. 1 which shows the distribution of standardised GRS and TEAM sum scores. Furthermore, the ranges and quartiles of all items (Additional file 3: Table S3) and the mean sum and mean GRS scores as percentages (Additional file 4: Table S4) are provided in the additional files.

### Sources of variation of TEAM scores across stations

In order to explain the variations in the overall TEAM scores (GRS) across cases, we estimated variance components and their relative contributions to the total variance using a mixed effects model (Table 3). The model includes random effects for raters, cases, rater-status (i.e., expert/novice) and team (i.e., the particular group of participants). We furthermore estimated random effects for the first-order interactions between cases and teams (do teams perform consistently across cases?) and cases and rater status (do experts and novices differ in their evaluations dependent on particular cases?). In total, the model accounted for 71.8% of the observed variance. We found that rater status (expert vs. novice) accounted for 11.1% of the variance of scores while the cases explained 10.2% of the variance. Teams accounted for 2.6% percent of variation in the observed scores while the biggest source of variance was the interaction of cases and teams with 43.2%, indicating that differences in scores were related to teams performing inconsistently across the different cases.

**Table 3:** Variance Components and Percentage of Variance for TEAM scores

Source of variance	Variance component	Percentage of variance
Rater^a^	0.048	1.32
Rater status^b^	0.397	11.05
Team	0.094	2.62
Case	0.366	10.17
Case × Team	1.553	43.21
Rater Status × Case	0.123	3.42
Residual	1.014	28.21

*Legend*: *TEAM* Teamwork Emergency Assessment Measure

^a^Rater includes all 12 raters. ^b^Rater status includes the categories ‘novice rater’ and ‘expert rater’

## Discussion

The aim of this study was to compare the rating behaviour of novices and experts using the previously established TEAM instrument. The idea to use novices to assess practical skills is not new, though we could find only one study that examined novices evaluating teamwork behaviour. Sevdalis and colleagues compared the ratings of an expert/expert pair to a novice/expert pair assessing surgical teamwork to analyse the construct validity of the OTAS tool and found relevant differences between expert and novice ratings on almost all items [46]. It is important to notice, though, that in this study the terms *expert* and *novice* referred to their experience in using the tool and both the two participating *experts* and the *novice* had backgrounds in psychology/human factors and were experienced in observing and rating behaviour. The present study, in contrast, defines experts and novices in terms of their content knowledge about teamwork and their practical experience. None of our raters had used TEAM before and they all received a rater training before the simulation.

When focussing on novice raters as raters who are new to or rather unexperienced in a certain area, the literature is generally in favour of novices (even students) being able to assess their peers, although the similarity to expert ratings depends on what skill is assessed and how [47–49]. A recent review [50] on peer assessment in objective structured clinical examinations (OSCE) showed that students awarded consistently higher ratings to their peers than experts when using GRS. Our study shows similar results when comparing the GRS scores, as novices rated the team behaviour on average 1 point higher than experts did (scale: 1–10); on some single items, novices rated significantly higher than experts, whereas in the majority of cases, including the sum score of all 11 items, there was no difference. In this context it is important to notice the large positive correlation of the sum scores of experts and novices as well as their consistent ratings of the best and worst performances, which justify the use of novices as raters. Novice raters’ tendency to give better ratings might be explained by a lower standard against which they compared their peers. Looking from the experts’ point of view, it seems plausible that experts are more aware of potentially serious consequences of bad teamwork because of their work experience and therefore rated more strictly [51, 52]. The moderate ICC of 0.66 is connected to this discrepancy between experts and novices. The 2 rater groups seem to have had different baselines, although all raters underwent the same training and anchoring process. The results of the *z* standardization of GRS and TEAM sum scores endorse this theory of different baselines. When each rater group’s scores were transformed to have a mean of 0 and a standard deviation of 1, their ratings showed very similar distribution patterns (similar range/interquartile range).

Unexpectedly, the teams themselves were only a very small source of the variance in performance scores (3%) and the interaction of team and case was by far the biggest source of variance (43%). In other words, a team’s performance varied considerably between the different cases and there were no superb or incapable teams per se. Importantly, since team leadership changed across cases, the 2 components (team leader and case) are confounded and thus cannot be disentangled statistically. Therefore, it is not clear whether variation in performances across cases is attributable to team leadership or the specific task. Still, our results suggest that a team’s performance depends to a considerable extent on the specifics of the situation. This finding has several implications. Firstly, it suggests that the recurrent finding of context specificity in clinical decision making of the individual is also relevant at the team level [53]. Secondly, this further emphasises the importance of a close investigation of what teamwork behaviour by whom is beneficial in exactly what situation—as opposed to generic rules meant to characterize ‘good teamwork’. Future training should abandon statements such as ‘practice closed-loop communication’ in favour of advice such as ‘During the first minutes of cardiopulmonary resuscitation (CPR), closed-loop communication initiated by the directive team leader is beneficial for CPR quality’ [6, 14]. Thirdly, TEAM scores should not be compared across different cases. The absence of clear benchmarks and the uncertain connection of TEAM scores and objective criteria remain problems when rating teams [25, 27].

As a beneficial side effect of our study, we validated the German version of TEAM, which is now available for clinical use (Additional file 5: Figure S1). Psychometric properties were comparable to those of the English original [24, 25, 27, 30, 31, 54] and the French translation [29]. The internal consistency for both novice and expert ratings was very high, the inter-rater reliability can be considered moderate, and the PCA confirmed 1 underlying component.

This study has several limitations. Firstly, it was a single-centre study with a small sample size. Although our number of observations (84) is similar to or even higher than in other studies using TEAM, our results are based on the ratings of 6 novice and 6 expert raters and each scenario was only observed by 2 of those 12 raters. Secondly, this study took place in a simulation setting that included different cases and changing team structure. Thirdly, our raters only observed monoprofessional teams, consisting of final year medical students. As our study is one of the first to use TEAM outside of typical resuscitation scenarios, more research is needed to decide how suitable TEAM is for rating teamwork behaviour in situations other than CPR and how to set performance benchmarks.

## Conclusions

Teamwork behaviour can be assessed with TEAM by novices just as well as by clinically experienced raters, though novices tend to rate slightly more lenient than experts do. Further research is needed on the comparability of TEAM scores across different cases. The German TEAM is a reliable and valid tool to assess teamwork performance that closes a gap in measuring teamwork behaviour in German-speaking countries.

## Additional files

Additional file 1: Overview of studies using TEAM including raters, ratees, and settings of these studies. (DOCX 24 kb)

Additional file 2: Cases and simulation settings (Discipline, diagnosis and mode of simulation of all 6 cases used in the study). (DOCX 17 kb)

Additional file 3: Range and quartiles of the 11 items of TEAM for novice and expert raters. (DOCX 21 kb)

Additional file 4: Means and standard deviations of sum and global rating scale scores and mean scores as percentages. (DOCX 14 kb)

Additional file 5: Team Emergency Assessment Measure (German translation). (DOCX 919 kb)

Additional file 6: Data Set (Team Emergency Assessment Measure scores of novices and experts rating 7 teams rotating through 6 cases each). (XLSX 15 kb)

## Abbreviations

CPR: Cardiopulmonary resuscitation
GRS: Global rating scale
ICC: Intraclass correlation coefficient
KMO: Kaiser–Meyer–Olkin
M: Mean
OSCE: Objective structured clinical examination
PCA: Principal component analysis
ROSC: Return of spontaneous circulation
SD: Standard deviation
TEAM: Teamwork Emergency Assessment Measure
TRAPD: Translation, review, adjudication, pre-testing, documentation

## Footnotes

1 In this study, we use the term *teamwork behaviour* to highlight that we treat non-technical skills such as communication and leadership skills at the team level as a kind of ‘collective’ non-technical skill; we did not evaluate team members individually.
