# Supplementary material for: Rating the quality of teamwork—a comparison of novice and expert ratings using the Team Emergency Assessment Measure (TEAM) in simulated emergencies
Source: Scand J Trauma Resusc Emerg Med. 2019 Feb 8;27:12. doi: 10.1186/s13049-019-0591-9 (PMC6368771; doi:10.1186/s13049-019-0591-9)
Supplement: Supplementary file 1 — Overview of studies using TEAM including raters, ratees, and settings of these studies. (DOCX 24 kb) [file 13049_2019_591_MOESM1_ESM.docx]

**Table S1** Overview of studies using TEAM including raters, ratees, and settings of these studies

| Study | Raters | Ratees | Setting/N |
| --- | --- | --- | --- |
| Cooper et al., Resuscitation, 2010  doi:10.1016/j.resuscitation.2009.11.027 | Part 1: *instrument testing*  S. Cooper, respectively S. Cooper and J. Porter (researchers; developers of TEAM) for inter-rater reliability | Hospital personnel; medical and nursing students (after attending a life support course) | *N* = 56; 3 hospital resuscitation events and 53 resuscitation simulations (all video recorded) |
|  | Part 2: *feasibility in “real time” events*  Experienced resuscitation trainers/clinicians | Second-year medical and nursing students (after attending a life support course) | *N* = 15 resuscitation simulations; teams of 5 students, TEAM rating directly after the scenario |
| Bogossian et al., Nurs Educ Today, 2013  doi:10.1016/j.nedt.2013.09.015 | Two trained clinicians | Final-year nursing students | *N* = 97 simulations with “deteriorating patients” (using SP), teams of 3 students; each team managed 3 scenarios with switching leaders |
| McKay et al., Resuscitation, 2012  doi:10.1016/j.resuscitation.2012.04.015 | Two clinical experts (resuscitation officer, anaesthetist) | Cardiac arrest teams (in total: 85 healthcare providers) | *N* = 24 resuscitation simulations (video recorded); 20 in simulation centre with small team (physician, anaesthetist, 2 nurses), 4 “in situ” in hospital with on-duty resuscitation team |
| Cooper et al., Emerg Med J, 2013  doi:10.1136/emermed-2012-201312 | Two clinical observers (researchers); one “only” observing, one participating in the simulation (playing an inexperienced doctor); ratings of first researcher were verified by second researcher after the scenario | Medical and surgical nurses | *N* = 44 simulations with “deteriorating patients” (using SP; acute myocardial infarction, hypovolaemic shock, chronic obstructive pulmonary disease); teams of 3 nurses, each team managing 3 scenarios with switching leaders |
| Cooper et al., Resuscitation, 2016  doi:10.1016/j.resuscitation.2016.01.026 | Forty-four senior registered nurses (resuscitation team members) | Resuscitation teams of 3 to 15 participants (raters were part of the teams) | *N* = 106 resuscitation events, setting: 2 Australian emergency departments, rating right after events |
| Maignan et al., Resuscitation, 2016  doi:10.1016/j.resuscitation.2015.11.024 | Nine raters with at least 2 years of experience in medical simulation | Ten teams of 4 care providers: 3 novice teams (13 fifth-year medical students), 4 intermediate teams (16 residents), 3 expert teams (11 certified emergency physicians); after attending an ALS course | *N* = 10 resuscitation simulations (video recorded; all 9 raters rated all 10 videos (in random order) for total of 90 observations |
| Cant et al., Emerg Med Australas, 2016  doi:10.1111/1742-6723.12643 | Nurses and medical staff (medical emergency team members); raters had an average of 5 years’ experience in emergency department | Medical emergency team members (same as raters) | *N* = 80 resuscitation events; at least 2 team members rated the TEAM for total of 283 observations, rating right after events; setting: 2 Australian emergency departments |

*Legend:* ALS = Advanced Life Support; SP = standardized patients; TEAM = Teamwork Emergency Assessment Measure.
