# Supplementary material for: Rating the quality of teamwork—a comparison of novice and expert ratings using the Team Emergency Assessment Measure (TEAM) in simulated emergencies
Source: Scand J Trauma Resusc Emerg Med. 2019 Feb 8;27:12. doi: 10.1186/s13049-019-0591-9 (PMC6368771; doi:10.1186/s13049-019-0591-9)
Supplement: Supplementary file 2 — Cases and simulation settings (Discipline, diagnosis and mode of simulation of all 6 cases used in the study). (DOCX 17 kb) [file 13049_2019_591_MOESM2_ESM.docx]

**Table S2** Cases and simulation settings

| Discipline | Diagnosis | Mode of simulation |
| --- | --- | --- |
| Pulmonology | Exacerbated COPD | SP, examination possible |
| Neurology | Ischaemic media-stroke | SP, examination possible |
| Cardiology | STEMI and non-sustained ventricular tachycardia | SP, examination possible |
| Anaesthesia | Ventricular fibrillation following STEMI | simulator-based approach |
| Traumatology | Haemodynamic instable ruptured spleen | simulator-based approach with advanced monitoring |
| Surgery | Head laceration with ethanol intoxication | SP, examination and preparation of wound possible |

*Legend:* COPD = chronic obstructive pulmonary disease; SP = simulated patient; STEMI = ST-elevation myocardial infarction.
