# Supplementary material for: Rating the quality of teamwork—a comparison of novice and expert ratings using the Team Emergency Assessment Measure (TEAM) in simulated emergencies
Source: Scand J Trauma Resusc Emerg Med. 2019 Feb 8;27:12. doi: 10.1186/s13049-019-0591-9 (PMC6368771; doi:10.1186/s13049-019-0591-9)
Supplement: Supplementary file 3 — Range and quartiles of the 11 items of TEAM for novice and expert raters. (DOCX 21 kb) [file 13049_2019_591_MOESM3_ESM.docx]

**Table S3** Range and quartiles of the 11 items of TEAM for novice and expert raters

| Item | Lower quartile (25th percentile) | | Median (50th percentile) | | Upper quartile (75th percentile) | | Range | |
| --- | --- | --- | --- | --- | --- | --- | --- | --- |
|  | N | E | N | E | N | E | N | E |
| 1: Leader gives directions/commands | 2 | 1 | 3 | 2 | 3.25 | 3 | 0–4 | 0–4 |
| 2: Leader maintains global perspective | 2 | 1 | 3 | 2 | 4 | 3 | 0–4 | 0–4 |
| 3: Team communicates effectively | 2 | 2 | 3 | 2 | 4 | 3 | 1–4 | 0–4 |
| 4: Team works together | 2 | 2 | 3 | 3 | 4 | 3 | 1–4 | 0–4 |
| 5: Team acts controlled | 2 | 2 | 3 | 3 | 4 | 4 | 1–4 | 0–4 |
| 6: Positive team morale | 2 | 3 | 3 | 3 | 4 | 4 | 1–4 | 1–4 |
| 7: Team adapts to changes | 2 | 2 | 3 | 3 | 3 | 3 | 1–4 | 0–4 |
| 8: Team monitors and reassesses | 2 | 2 | 3 | 2 | 4 | 3 | 1–4 | 1–4 |
| 9: Team anticipates actions | 2 | 2 | 3 | 2 | 3 | 3 | 0–4 | 1–4 |
| 10: Team prioritises tasks | 2 | 1 | 3 | 2 | 3.25 | 3 | 1–4 | 0–4 |
| 11: Team follows guidelines | 2 | 1 | 3 | 2 | 3 | 3 | 1–4 | 0–4 |
| Sum score | 22.75 | 19 | 31 | 27.5 | 37.25 | 33.25 | 14–44 | 6–43 |
| GRS score | 6 | 4.25 | 7 | 6 | 8 | 7.25 | 4–10 | 3–10 |

*Legend:* N = novice; E = expert; GRS = global rating scale.
