# Supplementary material for: Rating the quality of teamwork—a comparison of novice and expert ratings using the Team Emergency Assessment Measure (TEAM) in simulated emergencies
Source: Scand J Trauma Resusc Emerg Med. 2019 Feb 8;27:12. doi: 10.1186/s13049-019-0591-9 (PMC6368771; doi:10.1186/s13049-019-0591-9)
Supplement: Supplementary file 4 — Means and standard deviations of sum and global rating scale scores and mean scores as percentages. (DOCX 14 kb) [file 13049_2019_591_MOESM4_ESM.docx]

**Table S4** Means and standard deviations of sum and GRS scores and mean scores as percentages

| Measurement | Mean (SD) (score) | | Mean (percentage) | |
| --- | --- | --- | --- | --- |
|  | N | E | N | E |
| GRS score | 7.1 (1.6) | 6.1 (1.9) | 71 | 61 |
| Sum score | 30.4 (8.6) | 27.0 (8.4) | 61 | 61 |

*Legend:* SD = standard deviation; N = novice; E = expert; GRS = global rating scale.
