# Supplementary material for: Rating the quality of teamwork—a comparison of novice and expert ratings using the Team Emergency Assessment Measure (TEAM) in simulated emergencies
Source: Scand J Trauma Resusc Emerg Med. 2019 Feb 8;27:12. doi: 10.1186/s13049-019-0591-9 (PMC6368771; doi:10.1186/s13049-019-0591-9)
Supplement: Supplementary file 5 — Team Emergency Assessment Measure (German translation). (DOCX 919 kb) [file 13049_2019_591_MOESM5_ESM.docx]

**Team Emergency Assessment Measure (TEAM)**
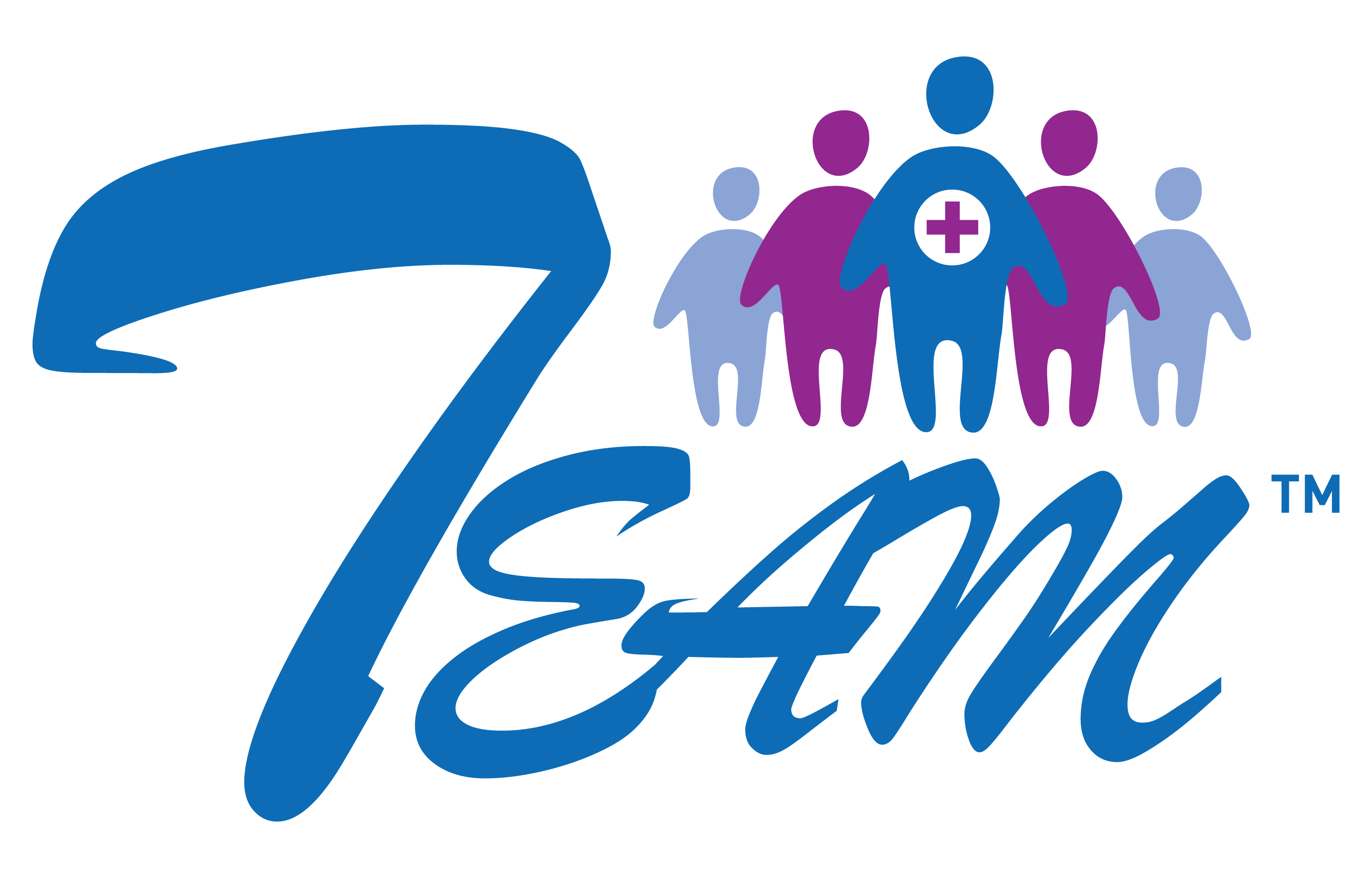


| **Einleitung** |
| --- |

Dieser Fragebogen zu nicht-medizinischen Fähigkeiten wurde als Beobachtungsbogen für die valide, reliable und praktikable Bewertung von notfallmedizinischen Teams (z.B. Reanimations- und Traumateams) entwickelt. Der Fragebogen sollte von erfahrenen Klinikerinnen und Klinikern ausgefüllt werden, um akkurate Performanzmessungen und Feedback zu Führungsrolle, Teamarbeit, zum Situationsbewusstsein und Aufgabenmanagement zu ermöglichen. Wo zutreffend, sind Hinweise zur Bewertung angegeben. Die folgende Skala liegt der Bewertung zugrunde:

| nie / fast nie | selten | ca. in der Hälfte der Fälle | oft | immer/fast immer |
| --- | --- | --- | --- | --- |
| 0 | 1 | 2 | 3 | 4 |

| **Angaben zum Team** |
| --- |

Datum: Uhrzeit: Ort: Teamleiter: Team:

| **Führungsrolle: Es wird angenommen, dass die Teamleitung entweder benannt ist, 0 1 2 3 4**  **aus der Situation entsteht oder die/der Erfahrenste ist - falls keine Teamleitung**  **besteht, vergeben Sie „0“ für Frage 1 und 2.** |
| --- |
| **1. Die Teamleitung ließ durch Anweisungen das Team wissen, was von ihm**  **erwartet wurde.** |
| **2. Die Teamleitung behielt eine globale Perspektive.**  *Hinweise: Überwachung klinischer Maßnahmen und der Umgebung? Versucht, wenn*  *möglich keine praktischen Aufgaben zu übernehmen (‘Hands off’)? Angemessene*  *Delegation von Aufgaben.* |
| **Teamarbeit: Bewertungen sollten (mehr oder weniger) das Team als Ganzes 0 1 2 3 4**  **umfassen, also Leitung und andere Mitglieder als Kollektiv.** |
| **3. Das Team kommunizierte effektiv.**  *Hinweise: Verbale, non-verbale und schriftliche Kommunikationsformen?* |
| **4. Das Team arbeitete zusammen um die Aufgaben zeitnah zu lösen.** |
| **5. Das Team agierte gefasst und kontrolliert.**  *Hinweise: angebrachte Emotionen? Probleme beim Konfliktmanagement?* |
| **6. Die Einstellung des Teams war positiv.**  *Hinweise: angemessene Unterstützung, Zuversicht, Stimmung, Optimismus,*  *Entschlossenheit?* |
| **7. Das Team passte sich an sich verändernde Situationen an.**  *Hinweise: Anpassung innerhalb der beruflichen Rolle?*  *Situationsänderung: Zustandsverschlechterung des Patienten? Veränderungen im Team?* |
| **8. Das Team überwachte und re-evaluierte die Situation.** |
| **9. Das Team antizipierte potentiell nötige Maßnahmen.**  *Hinweise: Vorbereitung der/s Defibrillators, Medikamente, Atemwegsmaterial?* |
| **Aufgabenmanagement: 0 1 2 3 4** |
| **10. Das Team priorisierte die Aufgaben.** |
| **11. Das Team folgte anerkannten Standards und Leitlinien.**  *Hinweise: Sind Abweichungen möglicherweise angebracht?* |
| **Gesamtleistung: 1 2 3 4 5 6 7 8 9 10** |
| **12. Vergeben Sie eine Gesamtbewertung für die nicht-medizinischen**  **Fähigkeiten des Teams auf einer Skala von 1-10** |

**Kommentare:______________________________________________________________________**

**_________________________________________________________________________________**
